# Insensitive Players? A Relationship Between Violent Video Game Exposure and Recognition of Negative Emotions

**DOI:** 10.3389/fpsyg.2021.651759

**Published:** 2021-05-21

**Authors:** Ewa Miedzobrodzka, Jacek Buczny, Elly A. Konijn, Lydia C. Krabbendam

**Affiliations:** ^1^Department of Communication Science, Faculty of Social Sciences, Vrije Universiteit Amsterdam, Amsterdam, Netherlands; ^2^Sopot Faculty of Psychology, SWPS University of Social Sciences and Humanities, Sopot, Poland; ^3^Department of Experimental and Applied Psychology, Faculty of Behavioural and Movement Sciences, Vrije Universiteit Amsterdam, Amsterdam, Netherlands; ^4^Department of Clinical Developmental Psychology, Faculty of Behavioural and Movement Sciences, Vrije Universiteit Amsterdam, Amsterdam, Netherlands

**Keywords:** video games, media violence, emotion recognition, empathy, adults, adolescents

## Abstract

An ability to accurately recognize negative emotions in others can initiate pro-social behavior and prevent anti-social actions. Thus, it remains of an interest of scholars studying effects of violent video games. While exposure to such games was linked to slower emotion recognition, the evidence regarding accuracy of emotion recognition among players of violent games is weak and inconsistent. The present research investigated the relationship between violent video game exposure (VVGE) and accuracy of negative emotion recognition. We assessed the level of self-reported VVGE in hours per day and the accuracy of the recognition using the Facial Expressions Matching Test. The results, with adolescents (Study 1; *N* = 67) and with adults (Study 2; *N* = 151), showed that VVGE was negatively related to accurate recognition of negative emotion expressions, even if controlled for age, gender, and trait empathy, but no causal direction could be assessed. In line with the violent media desensitization model, our findings suggest that higher self-reported VVGE relates to lower recognition of negative emotional expressions of other people. On the one hand, such lower recognition of negative emotions may underlie inaccurate reactions in real-life social situations. On the other hand, lower sensitivity to social cues may help players to better focus on their performance in a violent game.

## Introduction

The detrimental effects of playing violent video games have been a topic of debate for years, not only among policy makers and consumers (Nauroth et al., 2014), but also among scientists (e.g., Bushman et al., 2015; Ferguson and Konijn, 2015; Ivory et al., 2015; Teng et al., 2019; Coyne et al., 2020). The potential effect of playing violent, M-rated games on adolescents and adults is an important question to be studied for several reasons.

First of all, millions of people across the world are entertained by violent games on a daily basis (Boyle et al., 2012; Teng et al., 2019) and the most top-selling video games contain aggression and violence (Busching et al., 2015; Nielsen, 2015, 2016). Adolescents are the most avid users of digital games (Konijn et al., 2015; Coyne et al., 2020). In the United States 81% of adolescents had access to a gaming console (Lenhart, 2015) and the average gamer aged 13 and spends from 6.3 to 8.1 h a week playing video games (Nielsen, 2017).

Second, scholars argue pro and contra on how and why playing violent games may affect the psychological and social functioning of the players (Bushman and Huesmann, 2014; Elson and Ferguson, 2014; Ferguson and Konijn, 2015). Most of media violence research focused on studying effects on aggression (Anderson et al., 2010; Greitemeyer and Mügge, 2014; Chang and Bushman, 2019). Recently, problematic issues in these studies have been highlighted, for example, overestimated effect sizes in experiments and publication bias (Hilgard et al., 2017), challenging media violence effects on aggression. Moreover, media violence research often produced inconsistent results due to unstandardized behavioral measures of aggression (Elson et al., 2014; Ferguson et al., 2015). Finally, meta-analyses on the effects of violent video games and social behavior have yielded conflicting evidence. They showed either an influence of violent gaming on decreases in pro-social behavior (*r* = −0.11) and increases in aggression (*r* = 0.24 or *r* = 0.19; Anderson et al., 2010; Greitemeyer and Mügge, 2014, respectively), or close-to-zero effects both for pro-social (*r* = 0.04) and for aggressive behavior (*r* = 0.06 or *r* = 0.08; Ferguson, 2015; Prescott et al., 2018, respectively).

Promising ways to elucidate the effects of violent media use include: (1) studying cognitive and emotional mechanisms underlying aggression, (2) applying implicit measures preceding aggression, and (3) using better-standardized and more reliable instruments (cf. Ferguson and Konijn, 2015). Moreover, these methods can be used in experimental tasks in controlled laboratory environments. A relevant variable in this context is emotion recognition. The aim of the current research was to investigate the relationship between self-reported violent video game exposure (VVGE) and the accuracy of recognition of negative emotional expressions.

An important mechanism that may underlie aggression following violent media exposure is desensitization, defined as “a reduction in emotion-related physiological reactivity to violence” (Carnagey et al., 2007, p. 490). An integrated media violence desensitization processes model describes that being physiologically numb to violence affect cognitive and affective outcomes, for example, decreased sympathy for violence victims, and behavioral outcomes such as decreased helping or increased aggression (Carnagey et al., 2007; also demonstrated for VVGE in Bushman and Anderson, 2009). It could be argued that exposure to media violence may lead to altered processing of emotional information (Carnagey et al., 2007), including a lower sensitivity to social cues, which could be further observed in reduced recognition of emotional expressions. Based on the media violence desensitization model (Carnagey et al., 2007), we hypothesized that self-reported VVGE might be related to lower sensitivity to adequately recognize social cues, including emotional facial expressions.

It is important to research emotion recognition in the context of violent video game exposure since an ability to accurately recognize emotional expressions in others may have an impact on how players interact with the social world (e.g., North et al., 2010; Stanković et al., 2015). According to the “Violence Inhibition Mechanism” (Blair, 1995; Blair et al., 2001), the ability to identify distressing social cues, such as negative emotions, controls one’s behavior in response to other people’s emotional expressions. The expressions of negative emotions are also evolutionary more important than the expressions of positive states (Vaish et al., 2008) and negative emotions are considered to be more important than positive ones in evoking empathetic behavior (cf. Marsh and Blair, 2008). For instance, recognizing sadness or fear would trigger increased autonomic arousal, which would initiate avoidance or withdrawal that may help to limit the probability of violent behavior (Blair, 1995; Blair et al., 2001). Therefore, problems in recognizing negative emotions may have a negative impact on one’s social behavior. In line, impaired recognition of negative emotions was found as a common problem in adolescent offenders (Bowen et al., 2014; Gonzalez-Gadea et al., 2014), people high in trait psychopathy (Hastings et al., 2008) and in antisocial populations (Marsh and Blair, 2008).

### Violent Video Game Exposure and Emotion Recognition

Thus far, only a few studies explored the influence of exposure to violent games on the processing of facial expressions applying several approaches. In a dynamic emotion identification task, participants were asked to press a button on a keyboard when they noticed any change in a dynamic facial expression. Participants who were highly exposed to media violence were less sensitive to subtle changes in emotional expressions than the control group, low in media violence exposure (Kirsh et al., 2006). More specifically, the control group identified a change from neutral into a happy facial expression *faster* than a neutral face morphing to an angry facial expression, reflecting “the happy face advantage” effect (Kirsh et al., 2006). This effect was not found in the group high in violent media exposure – they identified angry facial expressions faster than happy faces (Kirsh et al., 2006). These results were further supported in a follow-up experiment: after 15 min of violent gameplay participants recognized angry emotional expressions faster than happy faces compared to those in the non-violent game condition (Kirsh and Mounts, 2007). In contrast, studies by Pichon et al. (2020) did not find a statistically significant relationship between habitual violent video gaming and accuracy in recognition of positive and negative facial expressions.

Another study investigating both accuracy and duration of emotion recognition suggested that associations with VVGE may depend on a specific emotional expression. Violent video game players recognized disgusted faces less accurately than non-gamers; however, they were better and faster in recognition of fearful emotional expressions than the control group (Diaz et al., 2016). Moreover, no differences were found for angry, happy, or sad faces (Diaz et al., 2016) between violent video game players and non-gamers.

Furthermore, two studies explored brain responses in an emotional search task in relation to VVGE. An event-related potentials (ERP) experiment showed that participants were slower identifying angry faces than happy faces after 25 min of violent video game exposure (Liu et al., 2017), which is inconsistent with the findings of Kirsh et al. (2006) and Kirsh and Mounts (2007). Moreover, violent gameplay resulted in a decreased N2pc amplitude for the happy faces, which reflects lower allocation of attention to these stimuli, as compared to the group playing a non-violent game (Liu et al., 2017).

This evidence was further extended by a longitudinal ERP study comparing effects of 10 h of either a violent or a non-violent video game training on the emotion search task. Findings of this study indicated that the violent game training resulted in a reduction in the ERP amplitude related to the allocation of attention to happy faces as compared to the condition before training; however, no changes were observed in angry faces processing (Bailey and West, 2013).

Additionally, two studies explored ERP responses to emotional faces in an inhibition task. An experimental study showed a decreased amplitude in early ERP components (N170 and P200) during emotional (happy or fearful) face processing after watching a violent movie (Stockdale et al., 2015). A cross-sectional study showed lower ERP amplitude to happy facial expressions in the frequent violent video game players group as compared to infrequent players; however, no difference was observed for fearful faces (Stockdale et al., 2017).

In sum, research shows that VVGE may be related to or may affect emotion recognition (Kirsh et al., 2006; Kirsh and Mounts, 2007; Bailey and West, 2013; Stockdale et al., 2015, 2017; Diaz et al., 2016). Most of the previous studies focused on how fast players could identify emotions or how they allocate attention to happy faces. The study by Diaz et al. (2016) examined the accuracy of recognition of emotional expressions providing mixed evidence, but the studies by Pichon et al. (2020) found not significant differences in accuracy of facial expressions recognition between habitual players of violent games and nonvideo game players. Based on that and the fact that ability to recognize negative emotions could prevent violent or anti-social behavior (Blair, 1995; Blair et al., 2001), the relationship between VVGE and recognition of negative emotions needs a further investigation. Moreover, earlier studies tested only adult participants, and to our knowledge, there is no study investigating the relationship between VVGE and emotion recognition in an adolescent sample. Studying adolescents is also important since this age group consists of the most avid users of digital games (Konijn et al., 2015; Coyne et al., 2020). In addition, adolescence is a sensitive period for social-cognitive development (Crone and Dahl, 2012), including emotion recognition (Thomas et al., 2007). Hence, exposure to violent video games may impact the ongoing development of adolescents social skills (Crone and Konijn, 2018; Stockdale and Coyne, 2018), such as recognition of emotional expressions.

### The Current Studies

The present two studies focused on accuracy of recognition of four expressions of negative emotions (anger, disgust, fear, and sadness) in relation to self-reported VVGE. The first study tested adolescents and the second one an adult sample. In both studies, we included male and female participants, and controlled for gender for two reasons. First, there are gender differences in emotion recognition (Montagne et al., 2005; Wells et al., 2016). Second, there are gender differences in video game preferences – boys play violent First-Peron Shooting (FPS) games more frequently than girls (Krahé and Möller, 2010; Rideout et al., 2010; Nije Bijvank et al., 2012; Wójcik, 2013). Finally, we also controlled for age of participants given developmental changes in emotion recognition (Thomas et al., 2007; Lee et al., 2013) and trait empathy, given that effects of self-reported VVGE on social outcomes may be partially mediated by empathy (Bartholow et al., 2005), and because females commonly exhibit higher empathy than males (for review see Christov-Moore et al., 2014).

## Study 1

The aim of this study was to investigate the relationship between exposure to violent games and recognition of negative emotions among adolescents. Specifically, we tested the hypothesis that a larger number of hours spent on violent gameplay per day would be related to less accurate recognition of negative emotional expressions in others.

### Method

#### Participants

Sixty-seven high school students (*M*_age_ = 17.06, *SD* = 0.69; age range: 16–18; 59.7% males; mainly Caucasians; details in Table 1) were recruited to voluntary participate in the study in one secondary school in Gdańsk, Poland. We ensured a safe climate and we believe that the participants’ motivation was genuinely intrinsic. The only incentive, we used was a snack that each participant could select afterward. No school representative obliged the adolescents to participate. The data collection was continued as long as the students were willing to volunteer.^1^

**Table 1:** Means, SD, and correlations between violent game time playing and additional variables in Study 1 (*N* = 67).

Variable	1	2	3	4	5	6
1. VVGE	–	−0.35^**^	−0.05	0.21	−0.01	−0.15
2. FEMT CORR		–	−0.02	−0.15	−0.28^*^	0.07
3. FEMT RT			–	0.13	0.10	0.10
4. Gender				–	−0.02	−0.24^*^
5. Age					–	−0.12
6. Empathy						–
*M*	0.82	64.05	4,336	–	17.06	3.53
*SD*	1.31	10.96	2,277	–	0.69	0.38

VVGE, violent video game exposure (in hours per day); FEMT CORR, correctness of emotion recognition in the FEMT (%); FEMT RT, mean reaction time of emotion recognition in the FEMT, 12 trials (in milliseconds); Gender: 1 = females, 2 = males; Empathy = trait empathy.

* *p* < 0.05

** *p* < 0.01.

According to a *post hoc* power analysis carried out in G*Power 3.1 (Faul et al., 2009), if a single predictor is inserted into a linear regression model, the current sample size of 67 participants has 0.72 power to detect an effect size of *f*^2^ = 0.10, 0.88 power to detect an effect size of *f*^2^ = 0.15, 0.95 power to detect an effect size of *f*^2^ = 0.20, and 0.98 to detect an effect size of *f*^2^ = 0.25. However, if four predictors are included (see Table 2), the current sample size of 67 participants has 0.48 power to detect an effect size of *f*^2^ = 0.10, 0.68 power to detect an effect size of *f*^2^ = 0.15, 0.82 power to detect an effect size of *f*^2^ = 0.20, and 0.90 to detect and effect size of *f*^2^ = 0.25. Based on Cohen (1988), it can be assumed that *f*^2^ = 0.10 is a small-to-medium effect size, *f*^2^ = 0.15 is a medium effect size, *f*^2^ = 0.20, and *f*^2^ = 0.25 are medium-to-large effect sizes (cf. G*Power, 2017, p. 19).^1^ However, for the limited added value of a *post hoc* power analysis, see Lakens (2021).

**Table 2:** Hierarchical multiple regression analyses predicting correctness of facial expression recognition as measured with the FEMT in Study 1 and Study 2.

	Study 1				Study 2			
	Δ*R*^2^	*B*	*SE*	*β*	Δ*R*^2^	*B*	*SE*	*β*
Step 1	0.02				0.04^*^			
Gender		−0.03	0.03	−0.15		−0.06	0.02	−0.21^*^
Step 2	0.01				0.01			
Gender		−0.03	0.03	−0.14		−0.05	0.03	−0.19^*^
Empathy		0.01	0.04	0.04		0.10	0.03	0.04
Step 3	0.08^*^				0.02^†^			
Gender		−0.04	0.03	−0.16		−0.05	0.03	−0.19^*^
Empathy		0.01	0.04	−0.01		0.02	0.03	0.06
Age		−0.05	0.02	−0.29^*^		0.01	0.01	0.14^†^
Step 4	0.11^**^				0.03^*^			
Gender		−0.20	0.03	−0.09		−0.02	0.03	−0.07
Empathy		−0.01	0.03	−0.04		0.02	0.03	0.07
Age		−0.05	0.02	−0.29^*^		0.01	0.01	0.12
VVGE		−0.03	0.01	−0.34^**^		−0.03	0.01	−0.21^*^
Total adj. *R*^2^	0.22^**^				0.10^**^			
*N*	67				151			

† *p* < 0.10

* *p* < 0.05

** *p* < 0.01.

Gender: 1 = females, 2 = males; Empathy = trait empathy; VVGE, violent video game exposure (in hours per day).

#### Procedure

The study protocol was approved by the Institutional Ethical Review Board. Students were randomly recruited from an information technology class to participate in a study about video games; participation was voluntary and anonymous.

The study was set up in a school library in conditions similar to a university lab. After entering the library, participants read and signed the informed consent form. Next, they completed the research procedure on a computer with the Inquisit (2013). First, they filled in questionnaires measuring traits: empathy, self-control (Self-Control Scale, SCS; Tangney et al., 2004; with corrections by De Ridder et al., 2011), and aggressiveness (the Buss-Perry Aggression Questionnaire, BPAQ; Buss and Perry, 1992). Then, participants did two tasks in randomized order: the Facial Expressions Matching Test (FEMT) and an emotional Stroop task (Dunajska et al., 2012), but the Stroop task is not part of the current study’s focus as emotional recognition and inhibitory control are not related and refer to different literatures.^2^ At the end, they answered questions about VVGE and demographic information. Upon completion, participants were debriefed and received a snack.

#### Measures

##### Emotion Recognition

**Figure 1:**
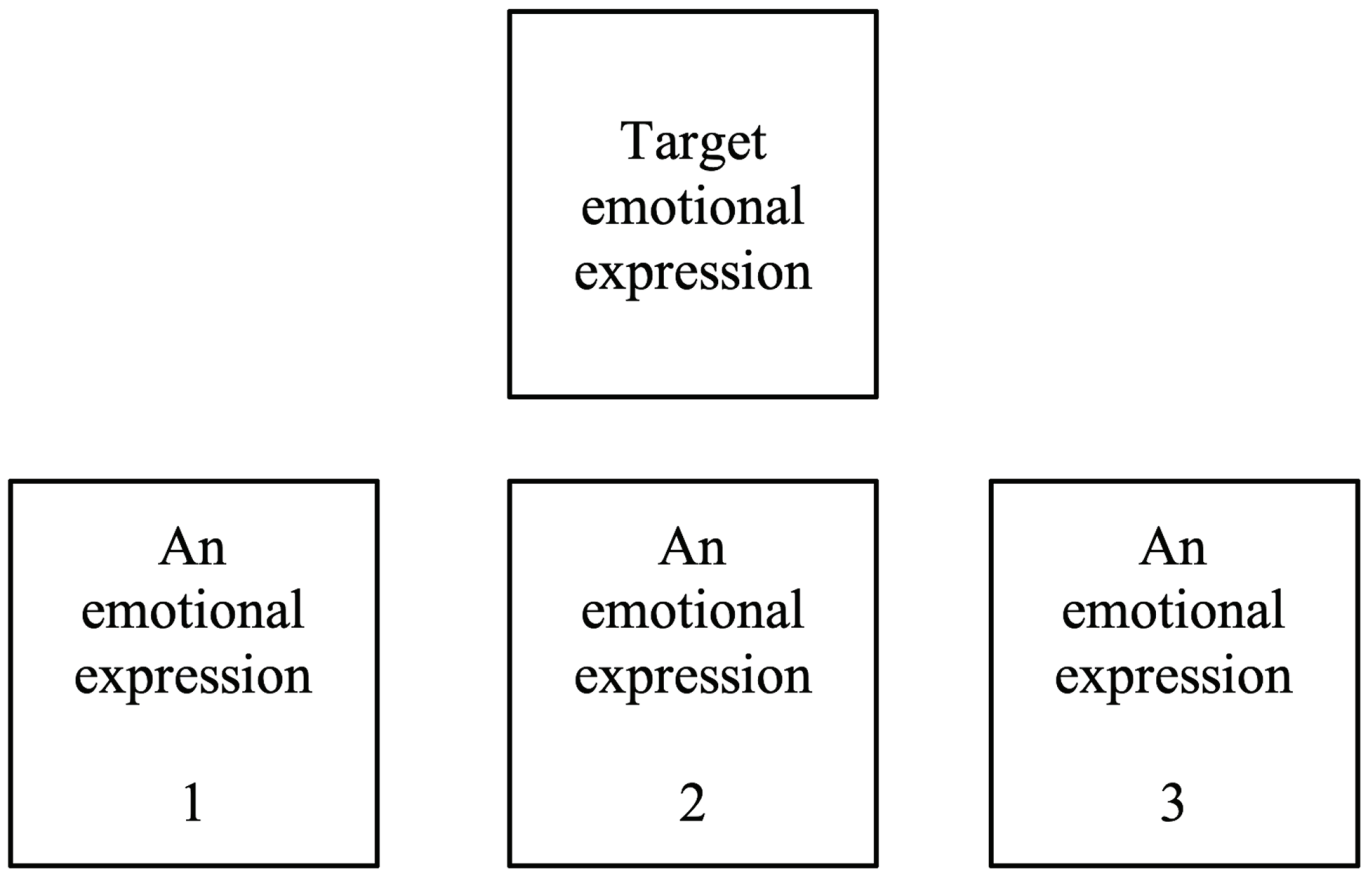
An example of one Facial Expressions Matching Test (FEMT) trial (for details see Szczygieł et al., 2012).

The FEMT (Szczygieł et al., 2012) is a computerized task that measures the accuracy of recognition of facial expressions of negative emotions: disgust, fear, anger, and sadness, presented by different actors, both males and females (following Ekman and Friesen, 1976; the stimuli can be viewed here: https://www.paulekman.com/product/pictures-of-facial-affect-pofa/). The FEMT enables a reliable and counterbalanced measurement of emotion recognition. Medium task difficulty (38–73%) allows testing recognition of negative emotions at different levels of this skill (Szczygieł et al., 2012, p. 434). In the FEMT, participants viewed screens displaying four faces. The task of a participant was to match one main emotion presented in the center of the computer screen with one of the other three emotional expressions presented at the bottom of the screen by pressing a button on a keyboard corresponding to a selected face. The stimuli were not randomized within each trial to avoid two types of problems: (1) the presentation of the same stimulus as the target and at the bottom of the screen, and (2) the impossibility to match expressions. We followed Szczygieł et al. (2012) configuration of pictures, which ensured that the location of each matching facial expression at the bottom of the screen and the order of trials were randomized and a face of the same person was not shown in the same trial (see Figure 1 for the structure of a FEMT trial; for details regarding the configuration, see Supplementary Material).

The FEMT consisted of 12 experimental trials that were preceded by two training trials. In total, participants viewed 56 pictures of emotional faces: eight in the training block, of which two were the target stimuli; and 48 in the second block, of which 12 were the target pictures. In the training block, participants received feedback on a screen if they chose a correct or incorrect answer. In the second block, participants received no feedback. Every experimental trial was randomized by the Inquisit 4 software and counterbalanced for each participant. Participants were instructed to give the most accurate and fastest responses in order to select their first choice. However, there was no time limit and each trial was displayed until a response was given. Therefore, the reaction times of emotion recognition (in milliseconds) are being treated as an additional measure in the FEMT. Correctness of facial expression recognition is considered as the main indicator in the FEMT. Each trial was coded in a way that there was always one correct answer possible. Correct answers were coded as 1, and incorrect answers were coded 0, which were further used to compute the percentage of correct answers (12 correct decisions = 100% correctness). Following the FEMT, we did not control for mood or any other potential confound of watching negative facial expressions because that was not relevant for our study’s goal.

##### Trait Empathy

The Interpersonal Reactivity Index (IRI; Davis, 1983; Kaźmierczak et al., 2007) is a 28-item questionnaire assessing empathy on three different dimensions: (1) Perspective Taking (PT): the tendency to spontaneously adopt the point of view of others; (2) Empathic Concern (EC): the ability to experience “other-oriented” feelings such as warmth and emotional compassion for people who are in unpleasant or painful situations; and (3) Personal Distress (PD): the tendency to experience “self-oriented” unpleasant emotions such as anxiety while observing suffering of others. The fourth dimension “Fantasy” in the original version by Davis (1983) was left out because this dimension captures an aspect of empathy less relevant to the current study, and given the age of the target group it was important to reduce the length of the questionnaire. Answers were given on five-point rating scales (1 = *does not describe me*, 5 = *describes me very well*). Cronbach’s *α* for the IRI was 0.81.

##### Exposure to Violent Video Games

Participants were asked several questions about their exposure to digital games: (1) “Do you play video games?”; (2) If the answer on the first question was “yes” – “How many hours per day did you play games in the last month?”; (3) “What kind of games did you play most often?”; and (4) “What is your favorite game?.” Based on the answers to these questions, and following guidelines of Busching et al. (2015) participants’ exposure to violent video games was measured. In order to assess the violent content of one’s favorite game, we used official ratings of the Pan European Game Information (PEGI).^3^ Moreover, we also included the amount of gameplay (i.e., average hours of gameplay per day), which is a common approach used in previous studies (Sobczyk et al., 2015; Diaz et al., 2016). Therefore, we used both hours of playing video games and the PEGI-ratings of one’s favorite game to measure VVGE. Based on that, self-reported VVGE was computed as a continuous variable, reflecting the number of hours per day spent on *violent* games (PEGI 18+, 16+, 12+ with a violent content label). The higher score on this rating, the higher exposure to violent video games in terms of more hours spent on violent gameplay per day. Participants, who reported at the first question that they do not play any games, automatically scored 0 in the violent exposure rating. Thus, the minimum value of the self-reported VVGE was 0 and the maximum in the current sample was 5 h.

### Results and Discussion

#### Analytical Approach

First, preliminary analyses provided information regarding participants’ VVGE and correlations between main variables of interest. Next, the main hypothesis was tested in a hierarchical regression analysis with the FEMT correctness as an outcome variable and gender, trait empathy, age, and self-reported VVGE (as a continuous variable, in hours per day) as predictors. In order to control for a potential effect of only a few influential cases, such as participants high in VVGE, a robust regression analysis was performed using R and MASS package (see Supplementary Material). The main purpose of the robust regression was to tone down the impact of a possible few influential cases (i.e., outliers) on the regression line (i.e., the relationship between VVGE and recognition of facial expression). Finally, we performed a *t*-test to compare groups: violent vs. non-violent video game players and non-players.

#### Preliminary Analyses

According to the VVGE measure, 41.8% of participants frequently played violent games (*n* = 28; *M* = 1.96, *SD* = 1.37 in hours per day), while 26 respondents (39%) did not play any video game at all and 13 responders played non-violent games (19%). Descriptive information and correlations between variables are presented in Table 1. A negative relationship between VVGE and the ability to recognize emotional expressions was observed (yet, note that there were many zero-scores which inflates correlations). A negative correlation was found between participants’ age and recognition of facial expressions. Females showed a higher level of trait empathy than males. Moreover, a negative relationship between VVGE and trait empathy was found. However, empathy was not related to the FEMT outcomes. Reaction times in the FEMT were not related to any other variable (see Table 1). Thus, analyses were controlled for these significant relationships.

#### Testing Hypothesis

In order to test the hypothesis that higher self-reported VVGE is negatively related to the correctness of facial expression recognition, hierarchical regression analysis, including gender in the first block, trait empathy in the second block, age in the third step, and self-reported VVGE (in hours per day) in the last step was performed. The order of the selected variables per block was determined to show that VVGE explains the variance of facial expression recognition (incremental validity) beyond relevant demographics (i.e., gender and age) or emotion-recognition-related traits (i.e., empathy).

**Figure 2:**
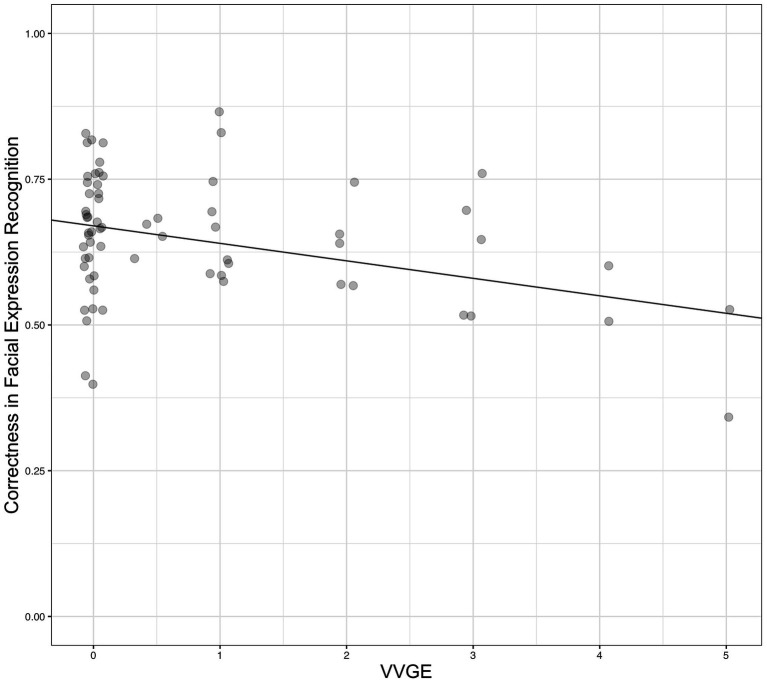
The relationship between violent video game exposure (VVGE; in hours per day) and correctness in recognition of facial expressions of negative emotions (FEMT; 1.00 = 100% correctness) in Study 1. The value VVGE = 0 represents non-violent gamers and participant who did not play any games, whereas the values VVGE > 0 represent players of violent games, *N* = 67.

Data from all participants were utilized to account for the differences in facial expression recognition in the sample. This analysis showed that VVGE and age were statistically significant predictors of correctness in the FEMT [full model: *F*(4, 62) = 4.24, *p* = 0.004, adj. *R*^2^ = 0.22].^4^ Thus, the current results supported our hypothesis. The effects of other covariates, i.e., gender and empathy, were statistically non-significant. Table 2 (left part) presents the details. In addition, the relationship between self-reported VVGE and facial expression recognition was presented in Figure 2.

#### Additional Analyses

We ran additional analyses with participants divided in two groups. The analyses showed that violent video game players (coded as 1) vs. the group of players of non-violent games and non-players (coded as 0) did not differ in accuracy and reaction times on the FEMT, age, or trait empathy (*t*s < 1.96). Significantly more males (22) than females (six) were violent video game-players, χ^2^(1, 67) = 7.12, *p* = 0.008, Cramer’s *V* = 0.33.

The results of the robust regression are presented in Supplementary Material. The analyses did not indicate to change the conclusion with respect to the test of the hypothesis.

In sum, these results indicate that exposure to violent video games (in hours per day) significantly relates to less accurate recognition of negative emotions in adolescent gamers, even when controlling for gender, age, and empathy. However, given the correlational nature, the direction of the relationship is not clear.

## Study 2

In the second study, we tested whether a similar hypothesis as for Study 1 with the adolescent sample, yet, in Study 2 with an adult sample. Thus, we expected that self-reported higher VVGE would be related to less accurate recognition of negative emotional expressions in others.

### Method

#### Participants

Adults were recruited at SWPS University in Poland to participate in the study (*N* = 151, *M*_age_ = 26.68, *SD* = 6.93; age range: 19–56, 28.5% males; mainly Caucasian; see details in Table 3). All participants agreed to participate voluntarily in the study. They received course credits for their participation. Given the sampling in a course context, we could not exclude females from participation. The data collection was continued as long as the students were willing to volunteer.^1^

**Table 3:** Means, SD, and correlations between violent game playing time and additional variables in Study 2 (*N* = 151).

Variable	1	2	3	4	5	6
1. VVGE	–	−0.27^*^	−0.12	0.51^***^	−0.12	−0.17^*^
2. FEMT CORR		–	0.27^**^	−0.21^*^	0.13	0.12
3. FEMT RT			–	0.07	0.29^**^	−0.02
4. Gender				–	0.03	−0.44^**^
5. Age					–	−0.15
6. Empathy						–
*M*	0.37	71.03	4,135	–	26.68	3.57
*SD*	0.98	12.69	2020	–	6.93	0.39

* *p* < 0.05;

** *p* < 0.01;

*** *p* < 0.001.

VVGE, violent video game exposure (in hours per day); FEMT CORR, correctness of emotion recognition in the FEMT (%); FEMT RT, mean reaction time of emotion recognition in the FEMT 12 trials (in milliseconds); Gender: 1 = females, 2 = males; Empathy = trait empathy.

As in Study 1, we calculated a *post hoc* power analysis using G*Power 3.1 (Faul et al., 2009) as reported above. For all assumed effect sizes and the given total sample size, the power was higher than 0.80.

#### Procedure and Measures

The study’s protocol was approved by the Institutional Ethical Review Board. The procedure was the same as in Study 1, including using the same measures as reported above. Upon arrival to the university’s laboratory, participants were informed that the study about was video games lasting for about 30 min. Participation was voluntary and anonymous. After signing the informed consent form, participants individually completed the research procedure on a computer using Inquisit (2013) software. After filling in the trait empathy questionnaire (IRI; Cronbach’s *α* = 0.78 for this study), SCS and BPAQ,^5^ participants completed two tasks in randomized order: the FEMT and emotional Stroop task.^2^ At the end, they answered questions about VVGE and provided demographic information. When finished, participants were debriefed.

### Results

Analyses were done in a similar way as in Study 1.

#### Preliminary Analyses

According to the VVGE responses, 17.2% of adult participants were regular violent game-players (*n* = 26; *M* = 2.15, *SD* = 1.32 in hours per day), and most of them did not have any experience with playing any type of video games (73.5%; *n* = 111). Only a few of them played non-violent games (9.3%; *n* = 14).

Descriptive information and correlations between variables are presented in Table 3. Self-reported VVGE negatively correlated with correctness of facial expression recognition. Males (coded as 2) had significantly higher self-reported VVGE than females (coded as 1). Furthermore, females were slightly better in recognition of emotions in the FEMT and presented higher levels of trait empathy than males. Reaction times in the FEMT were positively related to correctness of facial expression recognition and age, but were not related to gender nor trait empathy, and most importantly not to VVGE. Moreover, empathy was negatively related to self-reported VVGE.

#### Testing Hypothesis

**Figure 3:**
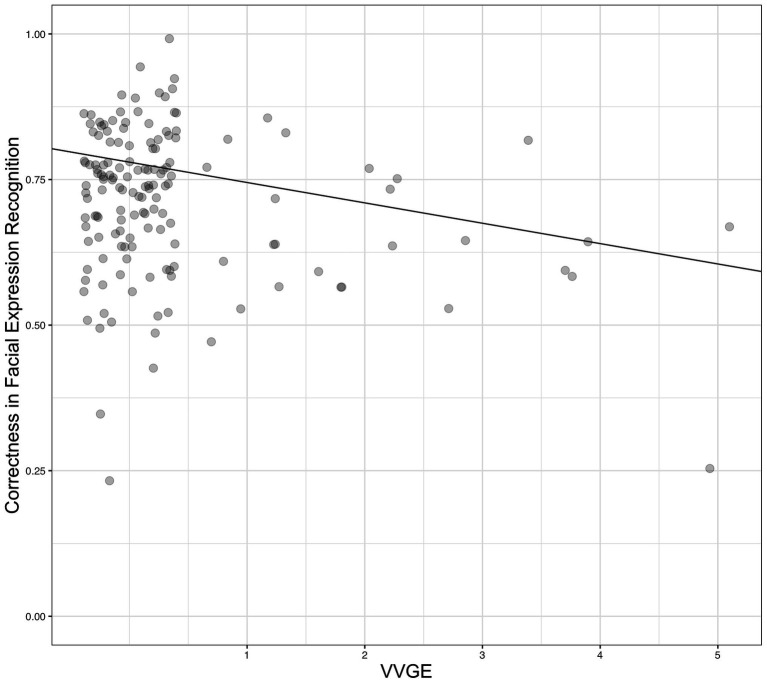
The relationship between VVGE (in hours per day) and correctness in recognition of facial expressions of negative emotions (FEMT; 1.00 = 100% correctness) in Study 2. The value VVGE = 0 represents non-violent gamers or participant who did not play any games, whereas the values VVGE > 0 represent players of violent games, *N* = 151.

In order to test the hypothesis that higher self-reported VVGE is negatively related to correctness of emotion recognition, we conducted a hierarchical regression analysis, similar to Study 1. Data from all participants were utilized to account for the differences in facial expression recognition among all participants. The results showed that VVGE was a statistically significant predictor of correctness of the FEMT, which corroborated our hypothesis.^6^ In the final model [*F*(4, 146) = 3.83, *p* = 0.005, adj. *R*^2^ = 0.10] the covariates were not significant but self-reported VVGE was statistically significant. Details are presented in Table 2 (right part). Figure 3 illustrates the relationship between VVGE and facial expression recognition in the adult sample.

#### Additional Analyses

We compared participants divided in two groups. The analysis showed that violent video game players (coded as 1) vs. the group of non-players and players of non-violent video games (coded as 0) did not differ in age nor in reaction times in the FEMT (*t*s < 1.96). However, the group of violent video game players showed significantly lower correctness in the FEMT [*t*(149) = 2.69, *p* = 0.008, Cohen’s *d* = 0.57; *M*_FEMT_ = 65.06%, *SD* = 12.24] and lower levels of trait empathy [*t*(149) = 2.83, *p* = 0.005, *d* = 0.61; *M*_empathy_ = 3.37, *SD* = 0.42] than the second group (*M*_FEMT_ = 72.27%, *SD* = 12.24; *M*_empathy_ = 3.60, *SD* = 0.38). In addition, significantly more males than females (22 vs. 4, respectively) were playing violent games, χ^2^(1, 151) = 48.60, *p* < 0.001, Cramer’s *V* = 0.57.

As in Study 1, the additional robust regression analyses (see Supplementary Material) did not change the conclusion regarding the test of the hypothesis in the current study.

In all, these results suggest that higher self-reported VVGE is related to less accurate recognition of negative emotion expressions. Moreover, both less accurate emotion recognition and lower level of trait empathy were observed in the group playing violent video games compared to the group consisting of non-violent gameplayers and non-players.

## General Discussion

The starting point for our research was the assumption that exposure to media violence might have a negative impact on processing of emotional stimuli (Kirsh et al., 2006; Kirsh and Mounts, 2007; Bailey and West, 2013; Diaz et al., 2016). Our findings supported the hypothesis that higher VVGE was negatively related to the accuracy of emotion recognition in others’ faces. Thus, the more hours per day adolescents and adults played violent video games, the less accurate they recognized the negative emotional expression, regardless of gender and individual differences in terms of trait empathy. However, this relationship might be reversed given the cross-sectional nature of the study in which no causal direction could be assessed.

The results were supported in two independent samples of participants. According to our knowledge, the present findings are the first suggesting worse recognition of negative emotions as related to self-reported VVGE, both in adolescents and adults. The more exposed a participant was to violent video games, the less accurate she/he was recognizing negative emotion expressions in images of other’s faces. Importantly, the comparison between the group of players of violent video games and the joint group of non-players and non-violent video game players showed no statistically significant differences in emotion recognition in Study 1. Therefore, we argue that exposure to violent video games shows an important inverse relationship with less accurate recognition of negative emotions by adolescents (Study 1), while both duration and violent content of video games play a role in those differences in adults (Study 2).

Our findings are in line with some previous studies showing that recognizing subtle changes in emotion expressions was more difficult for participants from a VVGE group than participants in a control condition (Kirsh et al., 2006; Kirsh and Mounts, 2007). However, in contrast to Kirsh et al. (2006), and Kirsh and Mounts (2007), we used a different method to asses emotion recognition and focused on accurate recognition of four negative emotions (anger, disgust, fear, and sadness), rather than on reaction times of emotion identification. Moreover, our findings add to current knowledge by showing that exposure to violent video games is related to recognition of four negative emotions, and not only disgust and fear (Diaz et al., 2016).

Our findings are further consistent with Plaisier and Konijn (2013), who argued that players of violent games may get trained to be self-focused and have difficulties in taking the perspective of others. Our results support this argument by showing that the more exposed individuals were to violent games, the worse they recognized facial expressions.

In our studies, we measured trait empathy and controlled for it in the analyses regarding the FEMT accuracy recognition. There is reason to expect that individuals high in trait empathy have better skills to identify a facial expression than individuals low in trait empathy (cf. Salovey and Mayer, 1990). This can be illustrated in psychopaths, who generally show low levels of empathy and use knowledge of other people’s emotions to manipulate them (Austin et al., 2007; Hansen et al., 2008). However, in both our studies, trait empathy was not significantly related to emotion recognition, whereas trait empathy was significantly and negatively related to the self-reported VVGE (only in the Study 2), which could partially support earlier findings (Anderson et al., 2010).

The association between self-reported VVGE and worse recognition of facial expression of negative emotions was observed both in adolescents and adult participants in the current studies. Importantly, age was significant related to the FEMT, but only in Study 1. It could be argued that the negative relationship between VVGE and negative emotions recognition could be more important for adolescents, who are in a sensitive period for social information processing (Crone and Dahl, 2012; Crone and Konijn, 2018). In a more general perspective, Stockdale and Coyne (2018) showed that young adult males were susceptible to video game addiction. Furthermore, the participants classified as addicts compared to non-addicts, showed stronger feelings of social isolation and lower emotional support.

In the adolescent sample, age was negatively related to the correctness of emotion recognition; thus, the older the participants were, the worse was their emotion recognition. However, since age differences in the adolescent sample were trivial, we do not interpret our results in terms of developmental changes related to age of participants. Moreover, because, we controlled for age in all our analyses, we ruled out the age factor as a possible alternative explanation in the differences of emotion recognition related to self-reported VVGE.

Previous studies showed poorer emotion information processing in violent video game players (Bartholow et al., 2006; Kirsh et al., 2006; Weber et al., 2006; Carnagey et al., 2007; Kirsh and Mounts, 2007; Bushman and Anderson, 2009; Engelhardt et al., 2011; Montag et al., 2012; Bailey and West, 2013), and their findings were understood as desensitization resulting from violent video gameplay. Desensitization refers to decreased experiencing of physiological emotion-related reactions. Although, we did not measure physiological reactions of participants in the present studies, our interpretation of the current results is consistent with the desensitization model: VVGE may be related to reduced sensitivity to social stimuli, which might become visible in worse recognition of others’ negative emotional states.

The findings of our cross-sectional studies are in line with previous experiments showing changes in lower sensitivity to emotional faces in players of violent video games (Bailey and West, 2013) and people who viewed a violent movie (Stockdale et al., 2015). Based on the mechanisms examined in the experimental studies, we suggest that frequent players of violent games in the current studies may be less sensitive to negative emotional expressions. While such lower sensitivity to negative emotional cues may have negative consequences in social situations, it may be beneficial in the context of violent video game environments where frequent players of video games were callous and used less cognitive resources to execute a specific task or action (Stockdale et al., 2017).

### Limitations and Future Directions

The studies have several limitations. Firstly, despite the consistent relationships found in the present studies, no causality can be inferred. The observed relationship could be either interpreted as a potential consequence of violent game playing or that people who already have problems in recognizing emotional expressions are more often playing violent video games. The current studies are exploratory in nature, and thus, more research (e.g., experiments and longitudinal) could test interdependence between developmental changes in emotion recognition ability and affinity for violent game playing. Importantly, future research should, in particular, focus on experiments that warrant an explanation of the processes underlying impaired recognition of emotional expressions associated with VVGE across age groups and cultures (cf. Teng et al., 2019), and use more objective measures of VVGE, instead of self-report, for example, similar to Johannes et al. (2021).

Secondly, our results are limited in the generalizability of the findings despite that we found a comparable relationship between self-reported VVGE and less accurate recognition of facial expressions in two independent samples (adolescents and adults) applying a validated method designed to measure behavioral outcomes (FEMT; Szczygieł et al., 2012). On the one hand, the outcomes have a relatively low level of statistical power and relatively small sample sizes that are not representative of the population of (violent) video gamers (including many non-gamers and more females than males). On the other hand, the effects we found in both samples were moderate (*β*’s > 0.20) when controlling for diverse covariates. In addition, we have carried out both studies in semi-controlled settings, which make a small-*N* design more acceptable (cf. Smith and Little, 2018). Finally, due to a relatively low number of regular violent game players in both studies (*n* = 28 and *n* = 26, respectively), and thus, due to the potential impact of a few influential cases (e.g., some participants playing violent games relatively more frequently), a robust regression analysis was performed. The results of the robust regression, weighting down the impact of the identified influential cases, provided similar support for the main conclusion derived from both studies regarding the relationship between VVGE and recognition of facial expressions. Nevertheless, to increase statistical power and generalizability, replications are needed with representative samples of adolescents and adults.

Thirdly, both studies differ in the proportion males-to-females. In Study 1, the gender ratio was closer to general population average (50–52%),^7^ whereas in Study 2, the percentage of females in the total sample was substantially higher than in the population. This further underscores the need for replication studies with samples that are more representative for video game players. Finally, the FEMT measures emotion recognition of four negative emotions. However, it does not allow computing accuracy of recognition for separate emotions due to the task design, where three different emotions are displayed at the same time. Therefore, we cannot directly compare our results with findings of Diaz et al. (2016), who found worse recognition for disgusted faces and better recognition for fearful faces related to exposure to violent video games. We consider the results we found as general for negative emotions. As the recent studies by Pichon et al. (2020) showed, accounting for the differences between facial expressions of various emotions or the magnitude of the expressions, could lead to a comprehensive view on the relationship between VVGE and recognition of emotion as expressed in the face. In this research, they did not find a statistically significant relationship between VVGE and recognition of facial expressions. However, in our studies, we used a slightly more sensitive measure of VVGE, whereas Pichon et al. (2020) dichotomized VVGE based on more complex criteria than in our studies. However, our study could not assess the direction of the relationship given the correlational nature. Thus, future studies could not only measure reactions to multiple types of facial expressions and their strength, but specifically also assess the direction of the relationship more carefully in experimental and longitudinal studies. Thereby also accounting for differences between adolescent and adult game players in the level of VVGE to be measured as a continuous variable, and preferably measured objectively. Because the relationship might also work the other way around, it is plausible to assume that the individuals who less accurately recognized facial emotion expressions than the others may have a prior preference to play violent games (Sauer et al., 2015), or may self-report the games as more violent (Przybylski and Weinstein, 2021) and spend more time playing violent games (Gao et al., 2017). Therefore, future studies could measure to what extent the (lower) ability to recognize emotions, or empathy in general, may precede violent gameplay, preference of violent games, and VVGE overall.

## Conclusion

The findings of our two studies showed that self-reported VVGE was related to lower recognition of negative emotional expressions of anger, sadness, fear, and disgust both in adults and in adolescents. However, the direction of the relationship could not be assessed given the study design and needs further inquiry. Problems with recognition of negative emotions in others may play an important role in guiding behavior of individuals frequently exposed to media violence, especially to violent video games. As a consequence, it may play a role in problems with real-life social interactions: not responding appropriately to social cues and inaccurate reactions toward other people. For example, it may lead to becoming insensitive, not identifying a suffering person in need, lowering chances of helping such a person.

## Data Availability Statement

The datasets presented in this study can be found in online repositories. The names of the repository/repositories and accession number(s) can be found at: https://osf.io/ma3ng/.

## Ethics Statement

The studies involving human participants were reviewed and approved by Komisja ds. Etyki Badań Empirycznych z Udziałem Ludzi jako Osób Badanych, Uniwersytet SWPS. Informed consents to participate in this study were collected from the participants (active consent). Participants’ legal guardian/next of kin were informed about the study (passive consent).

## Author Contributions

EM and JB developed the theory and method. EM collected the data. JB analyzed the results. All authors provided critical feedback and shaped the manuscript.

### Conflict of Interest

The authors declare that the research was conducted in the absence of any commercial or financial relationships that could be construed as a potential conflict of interest.

## Footnotes

^1^Since a meta-analysis of the studied relationship was not available, performing a sensible *a priori* power analysis was not possible. Thus, we could not estimate the required sample size based on a meta-analytical effect size beforehand.

^2^The analyses related to the additional measures (e.g., trait self-control) and Stroop task were not presented in the main article, but were included in Supplementary Material because they were not part of the current study and not relevant in relation to emotion recognition (i.e., emotion recognition and inhibitory control are not related and refer to different literatures). Data and R for selected analyses is available at https://osf.io/ma3ng/.

^3^www.pegi.info

^4^Because our sample size in Study 1 could be considered as small, and thus, our analyses could lead to false positives outcomes (cf. Wagenmakers et al., 2018), we performed an alternative analysis applying a Bayesian regression model in the statistical software jamovi (https://www.jamovi.org). Results showed that a model, which included VVGE and age as predictors provided the strongest support for H_1_ (BF_10_ = 51.69); averaged regression coefficient for VVGE was -0.026, 95% CI (-0.044, -0.009), for age was -0.039, 95% CI (-0.073, -0.007), *R*^2^ = 0.21, whereas a model, which included VVGE as the only predictor yielded substantially weaker support for H_1_ (BF_10_ = 11.38, *R*^2^ = 0.12), similarly for a model, which included all predictors (cf. Table 2) was analyzed (BF_10_ = 7.75, *R*^2^ = 0.22).

^5^Due to an error in part of an Inquisit executive script, only the BPAQ data were collected from 72 out of 151 participants in the total sample, whereas the data for other measures were complete.

^6^Again, we performed an alternative analysis applying a Bayesian regression model in the statistical software jamovi (https://www.jamovi.org). We found that a model, which included VVGE as the only predictor provided the strongest support for H_1_ (BF_10_ = 33.79); averaged regression coefficient was -0.03, 95% CI (-0.053, -0.013), *R*^2^ = 0.073, whereas a model, which included all predictors (cf. Table 2) yielded substantially weaker support for H_1_ (BF_10_ = 3.08, *R*^2^ = 0.095).

^7^https://ourworldindata.org/gender-ratio
