# Supplementary material for: Insensitive Players? A Relationship Between Violent Video Game Exposure and Recognition of Negative Emotions
Source: Front Psychol. 2021 May 21;12:651759. doi: 10.3389/fpsyg.2021.651759 (PMC8175673; doi:10.3389/fpsyg.2021.651759)
Supplement: Supplementary file 1 [file Table_1.DOCX]

Supplementary Material

# Supplementary Data

## Data of Study 1

<https://osf.io/6eyb5/>

## Data of Study 2

<https://osf.io/qgc4s/>

# Supplementary Code

## FEMT Codes (stimuli by Ekman & Friesen, 1976)

The matching facial emotional expression (the target and the appropriate facial expression) is annotated with *. The order of each trial was randomized. The location of the matching facial expression (presented at the bottom of the screen) was randomized, as well. The stimuli used in the training part and in the main part were fixed, as indicated below. Stimuli can be viewed here: <https://www.paulekman.com/product/pictures-of-facial-affect-pofa/>.

Training Trials

*1. 76 PE–5–07 At the bottom: 30 JB–1–23 15 EM–4–24* 16 EM–5–21

*2. 78 PE–3–16 At the bottom: 15 EM–4–24 37 JJ–5–13* 27 GS–2–25

Main Trials

*1. 37 JJ–5–13 At the bottom: 106 WF–3–4 76 PE–5–07 16 EM–5–21*

*2. 103 WF–5–6 At the bottom: 80 PE–2–21 40 JJ–3–20 15 EM–4–24*

*3. 16 EM–5–21 At the bottom: 59 MO–1–23* 2 A–2–06 108 WF–3–11

*4. 80 PE–2–21 At the bottom: 109 WF–4–22 30 JB–1–23* 37 JJ–5–13

*5. 27 GS–2–25 At the bottom: 30 JB–1–23 40 JJ–3–20* 78 PE–3–16

*6. 36 JJ–5–5 At the bottom: 67 NR–2–15* 3 A–1–14 25 GS–2–8

*7. 106 WF–3–4 At the bottom: 32 JB–1–16 80 PE–2–21* 37 JJ–5–13

*8. 25 GS–2–8 At the bottom: 59 MO–1–23* 43 JM–3–11 27 GS–2–25

*9. 36 JJ–5–5 At the bottom: 58 MO–1–30* 61 MO–2–11 16 EM–5–21

*10. 16 EM–5–21 At the bottom: 80 PE–2–21 103 WF–5–6 104 WF–3–16*

*11. 109 WF–4–22 At the bottom: 30 JB–1–23 40 JJ–3–20* 78 PE–3–16

*12. 15 EM–4–24 At the bottom: 106 WF–3–4 32 JB– 1–16 76 PE–5–07*

## Robust Regression for Study 1 and 2

R script: <https://osf.io/zfu46/>, R Markdown script: <https://osf.io/794dh/>.

## Figure for Study 1

R script: <https://osf.io/n5ctq/>, R Markdown script: <https://osf.io/8pt3q/>. The code generates Figure S1 (see below).


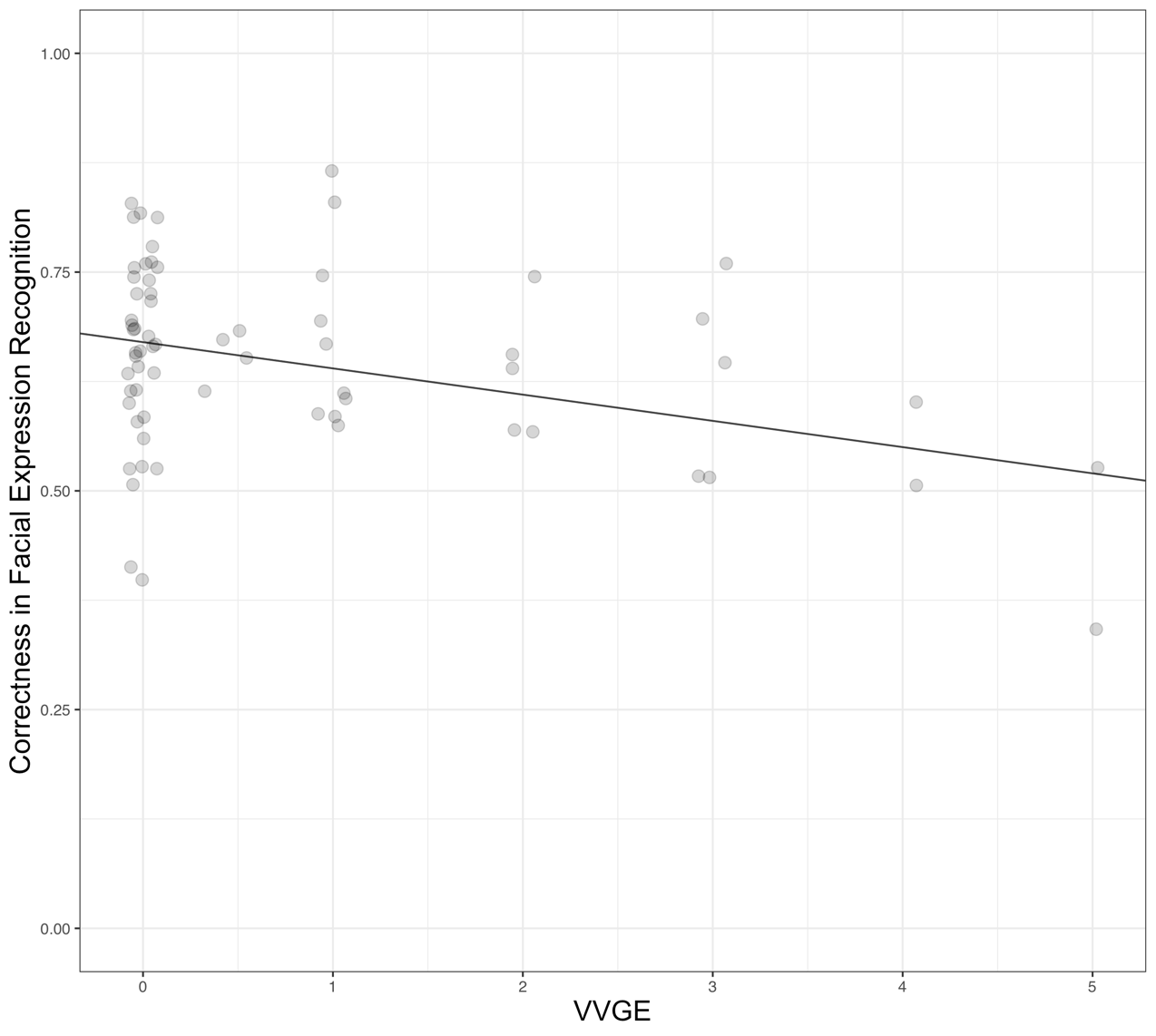


**Figure S1.** The relationship between violent video game exposure (VVGE; in hours per day) and correctness in recognition of facial expressions of negative emotions (FEMT; 1.00 = 100% correctness) in Study 1. The value VVGE = 0 represents non-violent gamers and participant who did not play any games, whereas the values VVGE > 0 represent players of violent games, *N* = 67.

## Figure for Study 2

R script: <https://osf.io/6vr2t/>, R Markdown: <https://osf.io/ueh5s/>. The code generates Figure S2 (see below).


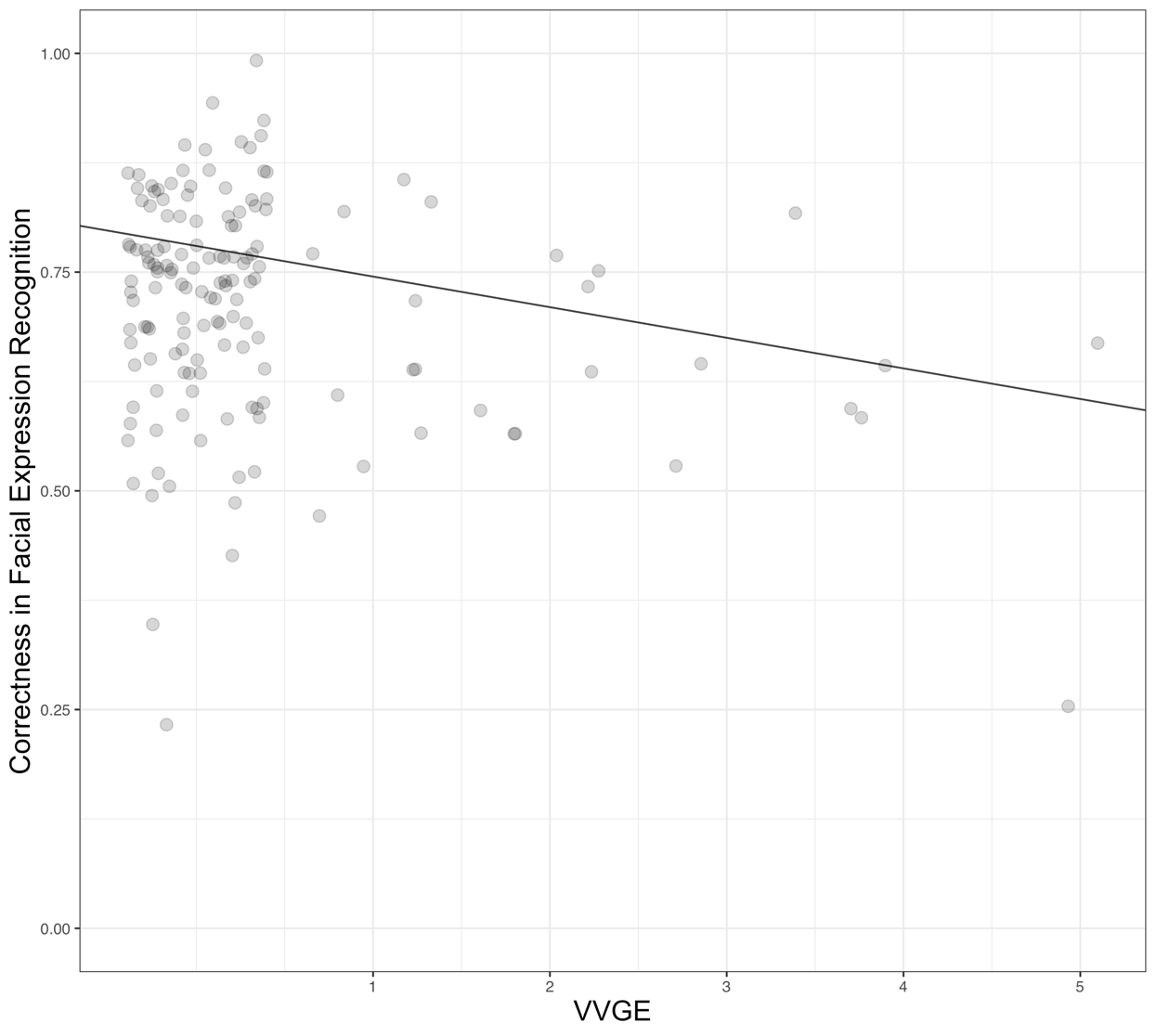


**Figure S2.** The relationship between violent video game exposure (VVGE; in hours per day) and correctness in recognition of facial expressions of negative emotions (FEMT; 1.00 = 100% correctness) in Study 2. The value VVGE = 0 represents non-violent gamers or participant who did not play any games, whereas the values VVGE > 0 represent players of violent games, *N* = 151.

# Supplementary Analyses

## Power Analysis for Study 1

*[1] –– Friday, March 19, 2021 –– 21:09:31*

**F tests** – Linear multiple regression: Fixed model, R² deviation from zero

**Analysis:** Post hoc: Compute achieved power

**Input:** Effect size f² = 0.10

α err prob = 0.05

Total sample size = 67

Number of predictors = 1

**Output:** Noncentrality parameter λ = 6.7000000

Critical F = 3.9885598

Numerator df = 1

Denominator df = 65

Power (1–β err prob) = 0.7224297

*[2] –– Friday, March 19, 2021 –– 21:09:42*

**F tests** – Linear multiple regression: Fixed model, R² deviation from zero

**Analysis:** Post hoc: Compute achieved power

**Input:** Effect size f² = 0.15

α err prob = 0.05

Total sample size = 67

Number of predictors = 1

**Output:** Noncentrality parameter λ = 10.0500000

Critical F = 3.9885598

Numerator df = 1

Denominator df = 65

Power (1–β err prob) = 0.8775927

*[3] –– Friday, March 19, 2021 –– 21:10:02*

**F tests** – Linear multiple regression: Fixed model, R² deviation from zero

**Analysis:** Post hoc: Compute achieved power

**Input:** Effect size f² = 0.20

α err prob = 0.05

Total sample size = 67

Number of predictors = 1

**Output:** Noncentrality parameter λ = 13.4000000

Critical F = 3.9885598

Numerator df = 1

Denominator df = 65

Power (1–β err prob) = 0.9501217

*[4] –– Friday, March 19, 2021 –– 21:10:15*

**F tests** – Linear multiple regression: Fixed model, R² deviation from zero

**Analysis:** Post hoc: Compute achieved power

**Input:** Effect size f² = 0.25

α err prob = 0.05

Total sample size = 67

Number of predictors = 1

**Output:** Noncentrality parameter λ = 16.7500000

Critical F = 3.9885598

Numerator df = 1

Denominator df = 65

Power (1–β err prob) = 0.9808456

*[5] –– Friday, March 19, 2021 –– 21:10:32*

**F tests** – Linear multiple regression: Fixed model, R² deviation from zero

**Analysis:** Post hoc: Compute achieved power

**Input:** Effect size f² = 0.10

α err prob = 0.05

Total sample size = 67

Number of predictors = 4

**Output:** Noncentrality parameter λ = 6.7000000

Critical F = 2.5201015

Numerator df = 4

Denominator df = 62

Power (1–β err prob) = 0.4852898

*[6] –– Friday, March 19, 2021 –– 21:10:54*

**F tests** – Linear multiple regression: Fixed model, R² deviation from zero

**Analysis:** Post hoc: Compute achieved power

**Input:** Effect size f² = 0.15

α err prob = 0.05

Total sample size = 67

Number of predictors = 4

**Output:** Noncentrality parameter λ = 10.0500000

Critical F = 2.5201015

Numerator df = 4

Denominator df = 62

Power (1–β err prob) = 0.6806794

*[7] –– Friday, March 19, 2021 –– 21:11:01*

**F tests** – Linear multiple regression: Fixed model, R² deviation from zero

**Analysis:** Post hoc: Compute achieved power

**Input:** Effect size f² = 0.20

α err prob = 0.05

Total sample size = 67

Number of predictors = 4

**Output:** Noncentrality parameter λ = 13.4000000

Critical F = 2.5201015

Numerator df = 4

Denominator df = 62

Power (1–β err prob) = 0.8174565

*[8] –– Friday, March 19, 2021 –– 21:11:10*

**F tests** – Linear multiple regression: Fixed model, R² deviation from zero

**Analysis:** Post hoc: Compute achieved power

**Input:** Effect size f² = 0.25

α err prob = 0.05

Total sample size = 67

Number of predictors = 4

**Output:** Noncentrality parameter λ = 16.7500000

Critical F = 2.5201015

Numerator df = 4

Denominator df = 62

Power (1–β err prob) = 0.9023092

## Power Analysis for Study 2

*[9] –– Friday, March 19, 2021 –– 21:11:53*

**F tests** – Linear multiple regression: Fixed model, R² deviation from zero

**Analysis:** Post hoc: Compute achieved power

**Input:** Effect size f² = 0.10

α err prob = 0.05

Total sample size = 151

Number of predictors = 1

**Output:** Noncentrality parameter λ = 15.1000000

Critical F = 3.9046281

Numerator df = 1

Denominator df = 149

Power (1–β err prob) = 0.9713321

*[10] –– Friday, March 19, 2021 –– 21:12:03*

**F tests** – Linear multiple regression: Fixed model, R² deviation from zero

**Analysis:** Post hoc: Compute achieved power

**Input:** Effect size f² = 0.15

α err prob = 0.05

Total sample size = 151

Number of predictors = 1

**Output:** Noncentrality parameter λ = 22.6500000

Critical F = 3.9046281

Numerator df = 1

Denominator df = 149

Power (1–β err prob) = 0.9971830

*[11] –– Friday, March 19, 2021 –– 21:12:13*

**F tests** – Linear multiple regression: Fixed model, R² deviation from zero

**Analysis:** Post hoc: Compute achieved power

**Input:** Effect size f² = 0.20

α err prob = 0.05

Total sample size = 151

Number of predictors = 1

**Output:** Noncentrality parameter λ = 30.2000000

Critical F = 3.9046281

Numerator df = 1

Denominator df = 149

Power (1–β err prob) = 0.9997672

*[12] –– Friday, March 19, 2021 –– 21:12:19*

**F tests** – Linear multiple regression: Fixed model, R² deviation from zero

**Analysis:** Post hoc: Compute achieved power

**Input:** Effect size f² = 0.25

α err prob = 0.05

Total sample size = 151

Number of predictors = 1

**Output:** Noncentrality parameter λ = 37.7500000

Critical F = 3.9046281

Numerator df = 1

Denominator df = 149

Power (1–β err prob) = 0.9999829

*[13] –– Friday, March 19, 2021 –– 21:12:34*

**F tests** – Linear multiple regression: Fixed model, R² deviation from zero

**Analysis:** Post hoc: Compute achieved power

**Input:** Effect size f² = 0.10

α err prob = 0.05

Total sample size = 151

Number of predictors = 4

**Output:** Noncentrality parameter λ = 15.1000000

Critical F = 2.4336334

Numerator df = 4

Denominator df = 146

Power (1–β err prob) = 0.8821889

*[14] –– Friday, March 19, 2021 –– 21:12:56*

**F tests** – Linear multiple regression: Fixed model, R² deviation from zero

**Analysis:** Post hoc: Compute achieved power

**Input:** Effect size f² = 0.15

α err prob = 0.05

Total sample size = 151

Number of predictors = 4

**Output:** Noncentrality parameter λ = 22.6500000

Critical F = 2.4336334

Numerator df = 4

Denominator df = 146

Power (1–β err prob) = 0.9772200

*[15] –– Friday, March 19, 2021 –– 21:13:02*

**F tests** – Linear multiple regression: Fixed model, R² deviation from zero

**Analysis:** Post hoc: Compute achieved power

**Input:** Effect size f² = 0.20

α err prob = 0.05

Total sample size = 151

Number of predictors = 4

**Output:** Noncentrality parameter λ = 30.2000000

Critical F = 2.4336334

Numerator df = 4

Denominator df = 146

Power (1–β err prob) = 0.9964635

*[16] –– Friday, March 19, 2021 –– 21:13:07*

**F tests** – Linear multiple regression: Fixed model, R² deviation from zero

**Analysis:** Post hoc: Compute achieved power

**Input:** Effect size f² = 0.25

α err prob = 0.05

Total sample size = 151

Number of predictors = 4

**Output:** Noncentrality parameter λ = 37.7500000

Critical F = 2.4336334

Numerator df = 4

Denominator df = 146

Power (1–β err prob) = 0.9995320

## Analyzes of all Variables in Study 1 and 2

The following analyzes include all measured variables. The main function Table S1 is to illustrate the effect of VVGE when recognition of facial expression was regressed on all variable.

**Table S1.** Hierarchical Multiple Regression Analyzes Predicting Correctness of Facial Expression Recognition as Measured with the FEMT in Study 1 and Study 2 with All Variables Included

|  | Study 1 | | | | Study 2 | | | |
| --- | --- | --- | --- | --- | --- | --- | --- | --- |
|  | Δ*R*^2^ | *B* | *SE* | β | Δ*R*^2^ | *B* | *SE* | β |
| Step 1 | .13 |  |  |  | .24* |  |  |  |
| Gender |  | –.031 | .028 | –.141 |  | –.091 | .035 | –.313* |
| Empathy |  | –.008 | .037 | –.027 |  | .057 | .043 | .158 |
| Age |  | –.047 | .020 | –.298* |  | .007 | .002 | .341* |
| TSC–IH |  | –.021 | .027 | –.131 |  | .008 | .025 | .039 |
| TSC–IN |  | –.009 | .024 | –.063 |  | .004 | .022 | .024 |
| Aggression |  | –.026 | .032 | –.116 |  | –.006 | .028 | –.026 |
| Step 2 | .10 |  |  |  | .06 |  |  |  |
| Gender |  | –.040 | .028 | –.180 |  | –.092 | .035 | –.315* |
| Empathy |  | –.009 | .038 | –.032 |  | .047 | .044 | .130 |
| Age |  | –.042 | .021 | –.265* |  | .007 | .002 | .356** |
| TSC–IH |  | –.033 | .027 | –.201 |  | .003 | .026 | .016 |
| TSC–IN |  | .006 | .024 | .043 |  | –.001 | .022 | –.005 |
| Aggression |  | –.032 | .031 | –.142 |  | –.002 | .028 | –.007 |
| StCneg |  | –.284 | .243 | –.152 |  | –.182 | .256 | –.083 |
| StCpos |  | .182 | .218 | .103 |  | –.428 | .245 | –.211 |
| StRTneg |  | .00016 | .00015 | .148 |  | .00003 | .0001 | .030 |
| StRTpos |  | –.0003 | .00015 | –.299* |  | .00001 | .0001 | .011 |
| Step 3 | .08* |  |  |  | .03 |  |  |  |
| Gender |  | –.026 | .027 | –.116 |  | –.056 | .041 | –.194 |
| Empathy |  | –.005 | .036 | –.019 |  | .038 | .044 | .106 |
| Age |  | –.043 | .020 | –.274* |  | .007 | .002 | .324* |
| TSC–IH |  | –.009 | .027 | –.056 |  | .006 | .025 | .029 |
| TSC–IN |  | .002 | .023 | .010 |  | –.007 | .022 | –.043 |
| Aggression |  | –.016 | .031 | –.074 |  | .00007 | .027 | .0003 |
| StCneg |  | –.162 | .238 | –.086 |  | –.191 | .252 | –.087 |
| StCpos |  | .241 | .210 | .137 |  | –.432 | .242 | –.213 |
| StRTneg |  | .00009 | .00014 | .085 |  | .00009 | .0002 | .081 |
| StRTpos |  | –.0003 | .00014 | –.277* |  | –.0001 | .0003 | –.005 |
| VVGE |  | –.027 | .011 | –.320* |  | –.031 | .020 | –.219^a^ |
| Total adj. *R*^2^ | .16** |  |  |  | .20^**^ |  |  |  |
| *N* | 67 |  |  |  | 72^a^ |  |  |  |

*Note*. **p* < .05. ***p* < .01.

Gender: 1 = females, 2 = males; Empathy = trait empathy; TSC–IH = trait self–control inhibitory; TSC–IN = trait self–control initiatory; Aggression = trait aggressiveness; StCneg = the difference between the correct responses in emotional Stroop test: negative trials minus neutral trials; StCpos = the difference between the correct responses in emotional Stroop test: positive trials minus neutral trials; StRTneg = the difference between reaction times in emotional Stroop test: negative minus neutral; StRTpos = the difference between reaction times in emotional Stroop test: positive minus neutral; VVGE = violent video game exposure (in hours per day).

^a^Due to an error in an Inquisit executive script (i.e., an entry to collect BPAQ data was not included in a part of the code where necessary), only BPAQ data were collected from 72 out of 151 participants in the total sample, whereas the data for other measures were complete. However, if aggression factor was excluded (*N* = 151) and the correctness of facial expression recognition was regressed on VVGE (Step 3) the effect of VVGE was: *B* = –.026, *SE* = .012, β = –.20*, adj. Δ*R*^2^ = .03*.

## Additional Regression Analysis in Study 1 and 2

Robust regression results can be found here: <https://osf.io/8qmr3/>.
